# Human T lymphotropic virus type-1 p30^II ^alters cellular gene expression to selectively enhance signaling pathways that activate T lymphocytes

**DOI:** 10.1186/1742-4690-1-39

**Published:** 2004-11-23

**Authors:** Bindhu Michael, Amrithraj M Nair, Hajime Hiraragi, Lei Shen, Gerold Feuer, Kathleen Boris-Lawrie, Michael D Lairmore

**Affiliations:** 1 Center for Retrovirus Research and Department of Veterinary Biosciences, The Ohio State University, Columbus, Ohio 43210, USA; 2 Department of Statistics, College of Mathematical and Physical Sciences, The Ohio State University, Columbus, Ohio 43210, USA; 3 Department of Microbiology and Immunology, State University of New York Upstate Medical University, Syracuse, New York 13210, USA; 4 Department of Molecular Virology, Immunology and Medical Genetics, The Ohio State University, Columbus, Ohio 43210, USA; 5 Comprehensive Cancer Center, The Arthur G. James Cancer Hospital and Solove Research Institute, The Ohio State University, Columbus, Ohio 43210, USA; 6 Department of Safety Assessment, Merck &Co., Inc. WP45-224, West Point PA 19486, USA

## Abstract

**Background:**

Human T-lymphotropic virus type-1 (HTLV-1) is a deltaretrovirus that causes adult T-cell leukemia/lymphoma and is implicated in a variety of lymphocyte-mediated disorders. HTLV-1 contains both regulatory and accessory genes in four pX open reading frames. pX ORF-II encodes two proteins, p13^II ^and p30^II^, which are incompletely defined in the virus life cycle or HTLV-1 pathogenesis. Proviral clones of the virus with pX ORF-II mutations diminish the ability of the virus to maintain viral loads *in vivo*. Exogenous expression of p30^II ^differentially modulates CREB and Tax-responsive element-mediated transcription through its interaction with CREB-binding protein/p300 and represses tax/rex RNA nuclear export.

**Results:**

Herein, we further characterized the role of p30^II ^in regulation of cellular gene expression, using stable p30^II ^expression system employing lentiviral vectors to test cellular gene expression with Affymetrix U133A arrays, representing ~33,000 human genes. Reporter assays in Jurkat T cells and RT-PCR in Jurkat and primary CD4+ T-lymphocytes were used to confirm selected gene expression patterns. Our data reveals alterations of interrelated pathways of cell proliferation, T-cell signaling, apoptosis and cell cycle in p30^II ^expressing Jurkat T cells. In all categories, p30^II ^appeared to be an overall repressor of cellular gene expression, while selectively increasing the expression of certain key regulatory genes.

**Conclusions:**

We are the first to demonstrate that p30^II^, while repressing the expression of many genes, selectively activates key gene pathways involved in T-cell signaling/activation. Collectively, our data suggests that this complex retrovirus, associated with lymphoproliferative diseases, relies upon accessory gene products to modify cellular environment to promote clonal expansion of the virus genome and thus maintain proviral loads *in vivo*.

## Background

Human T-lymphotropic virus type 1 (HTLV-1), the first characterized human retrovirus, causes adult T cell leukemia/lymphoma (ATL) and is associated with several lymphocyte-mediated disorders such as HTLV-1-associated myelopathy/tropical spastic paraparesis (HAM/TSP) [1]. Mature CD4+ T lymphocytes are the primary targets of HTLV-1 infection [2]. Although the mechanism by which the virus causes oncogenic transformation of host T lymphocytes is incompletely understood, altered gene expression has been associated with the initiation or progression of ATL [3]. This complex retrovirus encodes structural and enzymatic gene products, as well as regulatory and accessory proteins from open reading frames (ORF) in the pX region between *env *and the 3' long terminal repeat (LTR) of the provirus [4]. The well characterized Rex and Tax proteins are encoded in the ORF III and IV respectively. Rex is a nucleolus-localizing phosphoprotein, involved in nuclear export of unspliced or singly spliced viral RNA [5]. Tax is a nuclear and cytoplasmic localizing phosphoprotein that interacts with cellular transcription factors and activates transcription from the viral promoter, Tax-responsive element (TRE) and enhancer elements of various cellular genes associated with host cell proliferation [6]. Emerging evidence has documented the role of pX ORF I and II gene products in the replication of HTLV-1 [7,8]. There are four proteins expressed from these ORFs – p12^I^, p27^I^, p13^II^, and p30^II^. pX ORFs I and II mRNAs are present in infected cell lines and freshly isolated cells from HTLV-1-infected subjects [9], as well as in ATL and HAM/TSP patients [10]. Antibodies [11,12] and cytotoxic T cells [13] that recognize recombinant proteins or peptides of the pX ORF I and II proteins are present in HTLV-1 infected patients and asymptomatic carriers.

Using molecular clones of HTLV-1 with selective mutations of ORF I and II, we have tested the requirement of p12^I ^and p13^II^/p30^II ^in the establishment of infection and maintenance of viral loads in a rabbit model of infection [14-16]. ORF II protein p30^II ^contains a highly conserved bipartite nuclear localization signal (NLS) and localizes within the nucleus of cells [17-19]. In addition, p30^II ^contains serine- and threonine-rich regions with distant homology to transcription factors Oct-1 and -2, Pit-1, and POU-M1 [20]. Previous studies from our laboratory have demonstrated that p30^II ^also co-localizes with p300 in the nucleus and physically interacts with CREB binding protein (CBP)/p300 and differentially modulates cAMP responsive element (CRE) and TRE mediated transcription [18,21]. Recent reports also indicate a post-transcriptional role of HTLV-1 p30^II ^and HTLV-2 p28^II ^(homologous protein encoded in the HTLV-2 pX ORF II region), in modulating the export of tax/rex RNA from the nucleus [22,23]. Therefore, p30^II ^appears to be a multi-functional protein with transcriptional and post-transcriptional roles in regulating viral gene expression. Based on these reports, we hypothesized that p30^II ^functions as a regulator of cellular and viral gene expression to promote HTLV-1 replication.

Gene arrays have primarily been employed to study the changes in gene expression profile of HTLV-1-immortalized and transformed cell lines or in cells from ATL patients and attempts to test the influence of individual HTLV-1 viral proteins on cellular gene expression have been limited to Tax [3,24-27]. Herein we used the Affymetrix U133A human gene chip to confirm the role of p30^II ^as a regulator of gene expression and identified several novel and important alterations in gene expression profiles, unique to cell cycle regulation, apoptosis and T cell signaling/activation. In addition, using semi-quantitative RT-PCR, we have confirmed the expression of multiple genes modulated by p30^II ^in Jurkat T cells and primary CD4+ T lymphocytes. We then tested the influence of p30^II ^in T cell signaling using reporter assays representing critical T lymphocyte transcription factors. This is the first report that demonstrates the role of p30^II ^as an activator of key transcription factors involved in T cell signaling/activation. Together, our data suggests that HTLV-1, a complex retrovirus associated with lymphoproliferative disorders, uses accessory genes to promote lymphocyte activation to enhance clonal expansion of infected cells and maintain proviral loads *in vivo*.

## Results

### p30^II ^and Analysis of Cellular Gene Expression in Jurkat T Lymphocytes

**Figure 1:**
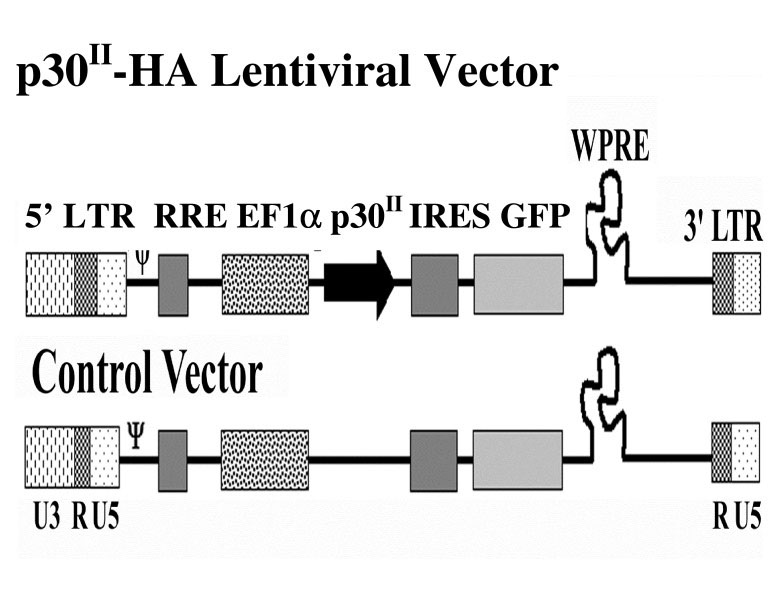
**Schematic illustration of lentiviral vectors expressing both p30HA and GFP (sample vector) as bicistronic messages and GFP alone (control vector) from elongation factor 1 alpha promoter. **Abbreviations: LTR – Long Terminal Repeats; RRE – Rev Response Element; EF1 α – Elongation Factor 1 alpha promoter; IRES – Internal Ribosome Entry Site; WPRE – Woodchuck Hepatitis Post-transcriptional Regulatory Element.

**Figure 2:**
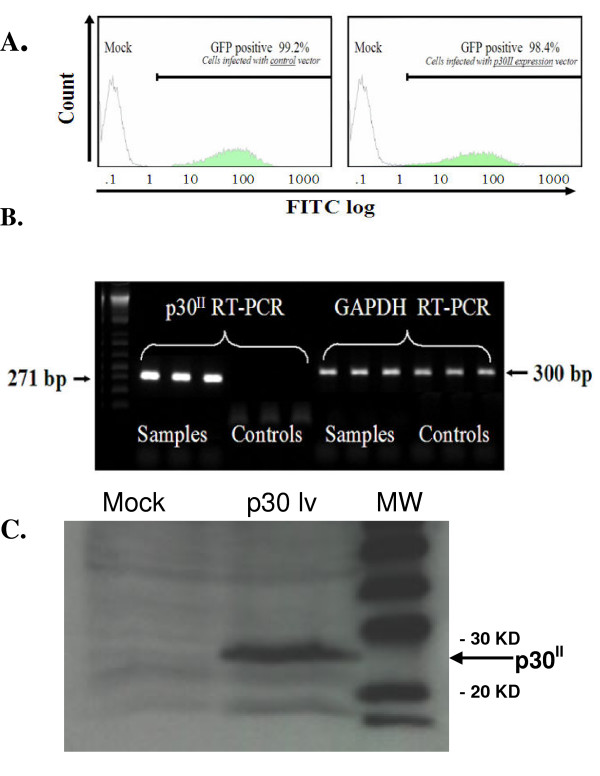
**Triplicate p30^II ^samples express GFP and p30^II ^while triplicate controls express only GFP. **(A) Flow cytometric analysis illustrating the expression of GFP in Jurkat T cells 10 days post spin-infection with lentiviral vectors. Both sample (expressing p30^II ^and GFP) and control (GFP alone) group contains relatively high and similar levels of GFP. (B) RT-PCR demonstrating the expression of p30^II ^in Jurkat T cells 10 days post spin-infection with lentiviral vectors. Jurkat T cells spin-infected with sample vector express p30^II ^while the control vector spin-infected cells do not express p30^II^. RT-PCR was performed with triplicate samples and controls. GAPDH was used as a control for the integrity of the message. (C) Representative western blot showing p30^II ^expression from cell lysate (p30^II ^migrates at ~28 kD). M = Mock vector infected cell lysate, p30 lv = p30 lentivirus vector infected cell lystate, MW = biotin molecular weight markers.

Stable expression of HTLV-1 p30^II ^in Jurkat T lymphocytes was established using recombinant lentiviruses (Fig. 1). At 10 days post-transduction, GFP expression was greater than 95% in Jurkat T lymphocytes transduced with recombinant lentivirus expressing GFP alone (controls) or p30^II ^and GFP (samples) (Fig. 2). RT-PCR was used to confirm the expression of p30^II ^mRNA in the sample cells and absence of p30^II ^mRNA expression in control cells (Fig. 2). p30^II ^protein expression was also confirmed by western immunoblot assay (data not shown) using methods as previously reported [28]. Differential gene expression and comparative analysis was done to identify probes with at least 1.5 fold difference in expression between control and p30^II ^and verified for cluster formation [29]. Quality control criteria evaluations included comparison of the ratios of 3' signal to 5' signal of two housekeeping genes, beta-actin and GAPDH, which were between 0 and 3. Additional hybridization controls were used in each array and included BioB, BioC, BioD, and Cre. These controls were all present and in a linear relationship of intensity. Quantitative RNA levels were determined by comparing the average differences representing the perfectly matched minus the mismatched for each gene-specific probe set before analysis with data mining software to identify probes with at least 1.5 fold differences [29,30].

We then categorized genes deregulated by HTLV-1 p30^II ^into those upregulated or downregulated in expression. We further grouped genes deregulated by p30^II ^based on their functions, such as apoptosis, cell cycle, cell adhesion, transcription/translation factors and T cell activation or cell signaling. In all the categories, p30^II ^was an overall repressor of cellular gene expression, while selectively increasing the expression of certain key regulatory genes (Table 1, see Additional file 1). The total number of genes of known biological or molecular function that were decreased in expression was 318 compared to 126 genes that were increased in expression.

### p30^II ^Modulates Multiple Cellular Gene Networks

Based on changes in gene expression in p30^II ^expressing cells (Table 1), p30^II ^would be predicted to modulate apoptosis. These include Bcl-2 related/interacting genes such as anti-apoptotic Bcl-2-related protein A1, anti-apoptotic MCL1, cell-death regulator Harakiri, apoptotic protector BNIP1 (downregulated) and pro-apoptotic BIK (upregulated). In addition, p30^II ^expression correlated with downregulation of genes associated with Fas mediated apoptosis pathway such as tumor suppressing subtransferable candidate 3 and TNF receptor superfamily member 25. p30^II ^expression was also associated with decreased expression of caspases (2 and 4) and increased expression of genes associated with the DNA fragmentation pathway (CIDE-B and CIDE-3). In addition, p30^II ^expression correlated with decreased expression of many other apoptosis related genes including CD28, Lck, cyclin B1, Cullin 5, Adenosine A2a receptor, TAF4B and NCK-associated protein 1.

Multiple genes involved in cell cycle regulation were altered in p30^II ^expressing Jurkat T lymphocytes. These include checkpoint suppressor 1, cytosolic branched-chain amino acid transaminase 1, histone deacetylase 6, cyclin B1, WEE1 kinase, CDC14A, Lck, JAK2, GAS7, BZAP45, Cullin, Rab6 GTPase activating protein (downregulated) and TERF1, AKAP8, DDX11, MSH2 and JUN-D (upregulated). Another gene down regulated by p30^II ^expression was MDM2, which is over expressed in certain types of leukemia [31] and capable of enhancing the tumorigenic potential of cells by inhibiting p300/PCAF mediated p53 acetylation [32].

p30^II ^expression was associated with altered expression of several genes involved in cell-to-cell adhesion. These include decrease in integrin (integrin β8) immunoglobulin (MADCAM1), a counter-receptor for P-selectin (SELPLG), cadherin (desmocollin 3), protocadherin (PC-LKC) liprin (PPF1BP1), CD84/Ly-9, CD58, CD43/sialophorin and glycosyl-phosphatidyl-inositol phospholipase D1. Expression of p30^II ^correlated with increase in integrin receptor α1 subunit and KIT ligand.

A number of genes encoding transcriptional control factors or regulators of transcription were repressed in p30^II ^expressing Jurkat T lymphocytes. These included decreased expression of TATA-binding protein associated factor 4 (TAF4), two co-repressors (Enolase-1 and Chromosome 19 ORF2 protein), a novel specific coactivator for mammalian TEFs, namely TONDU [33], homeo box genes (mesenchyme homeo box 1, homeobox A1), T-box genes (T-box 21) and proteins containing helix-loop-helix domain, which are known to be critical in cell growth/differentiation and tumorigenesis (neuronal PAS domain protein 2, Myc-associated factor protein, inhibitor of DNA binding-3). Additionally, p30^II ^expression correlated with down regulation of zinc finger proteins (zinc finger protein 36), a group of transcription regulators proposed to be candidates in malignant disorders [34] and coiled coil proteins (JEM-1). p30^II ^was also associated with downregulation of many genes with positive transcriptional effects (including SEC14-like 2, Nurr 1, CITED2/MRG1, LXR alpha and SMARCA2). Reduced expression of HDAC6, a histone deacetylase and nuclear receptor coactivator 3 (CBP interacting protein) with histone acetyltransferase and pCAF/CBP recruiting abilities [35] are particularly interesting, since p30^II ^contains multiple highly conserved lysines, which could play a role in acetylation [18,21]. Expression of p30^II ^was also associated with decrease in GAS 7, which has sequence homology to Oct and POU family of transcription factors [36] and decreased expression of translation initiation factor 2 (IF2) and eukaryotic translation elongation factor 1δ (EEF1D). In contrast, p30^II ^expression in Jurkat T lymphocytes was associated with an increase in expression of eukaryotic translation elongation factor 1α (EEF1A2), a putative oncogene [37], and enhanced expression of HTLV enhancer factor, Jun-D, TAF1C, Kruppel-type zinc finger, PQBP1, AF4 and SOX4.

### p30^II ^Expression Alters Patterns of T-Cell Signaling Gene Networks

**Figure 3:**
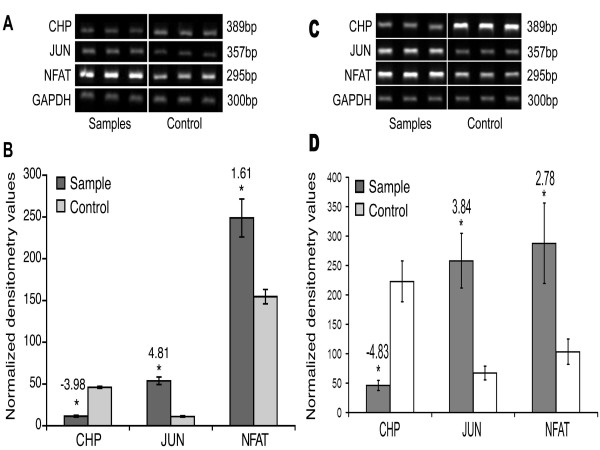
**Semiquantitative RT-PCR of CHP, JUN-D and NFATc in controls and p30^II ^expressing Jurkat T lymphocytes (A and B) and primary CD4+ T lymphocyte (C and D) samples. **PCR products were separated by electrophoresis (A and C), normalized to GAPDH and quantified by densitometry (B and D). In panel B, dark grey bars indicate indicate p30^II ^expressing cells and light grey bars indicate control (empty vector) cells. In panel D, dark grey bars indicate indicate p30^II ^expressing cells and white bars indicate control (empty vector) cells. Data points are mean of triplicates. CHP was downregulated while JUN-D and NFATc was upregulated by p30^II^. Fold decrease/increase in activity in the presence of p30^II ^are indicated above each bar.

Genes involved in T-cell signaling were differentially affected by p30^II ^expression. Expression of p30^II ^was associated with decreased expression of CD28, a co-stimulatory molecule with a distinct role in T lymphocyte activation [38] and reduced gene expression of CD46 and Lck tyrosine kinase, a member of the Src family of tyrosine kinases activated by T cell surface receptors [39]. In contrast, cells expressing p30^II ^had enhanced Vav-2 and CD72 gene expression. Additionally, p30^II ^expression correlated with decrease in the level of CHP, an endogenous calcineurin inhibitor, which would be predicted to promote NFAT expression by p30^II ^(see below). Moreover, p30^II ^expression was associated with increased expression of Jun-D and c-Fos, suggesting activation of AP-1 mediated transcription. p30^II ^expression was associated with decreased expression of protein kinase D (PKD), which negatively modulates JNK signaling pathway [40], mediates cross-talk between different signaling systems, and is critical in processes as diverse as cell proliferation and apoptosis [41]. Interestingly, in Jurkat T lymphocytes expressing p30^II^, there were no detectable levels of I kappa B kinase gamma (IKKγ), which is important for NF-κB signaling in response to both T cell activation signals and Tax [42]. p30^II ^expression was associated with increased Hematopoetic Progenitor Kinase-1 (HPK-1), a known NF-κB activator [43]. p30^II ^expression was also associated with decreased Ras GRP2, a guanyl nucleotide exchange factor that increases Ras-GTP, suggesting a decrease in the level of activated Ras (Ras-GTP). Seminquantitative RT-PCR analysis in Jurkat T lymphocytes and primary CD4+ T lymphocytes correlated directly with the gene array and confirmed the altered expression of each of three selected genes involved in these T cell activation/signaling pathway (Fig. 3A through 3D).

### p30^II ^Influences NFAT, NF-κB and AP-1-mediated Transcription in Co-Stimulated Jurkat T lymphocytes

**Figure 4:**
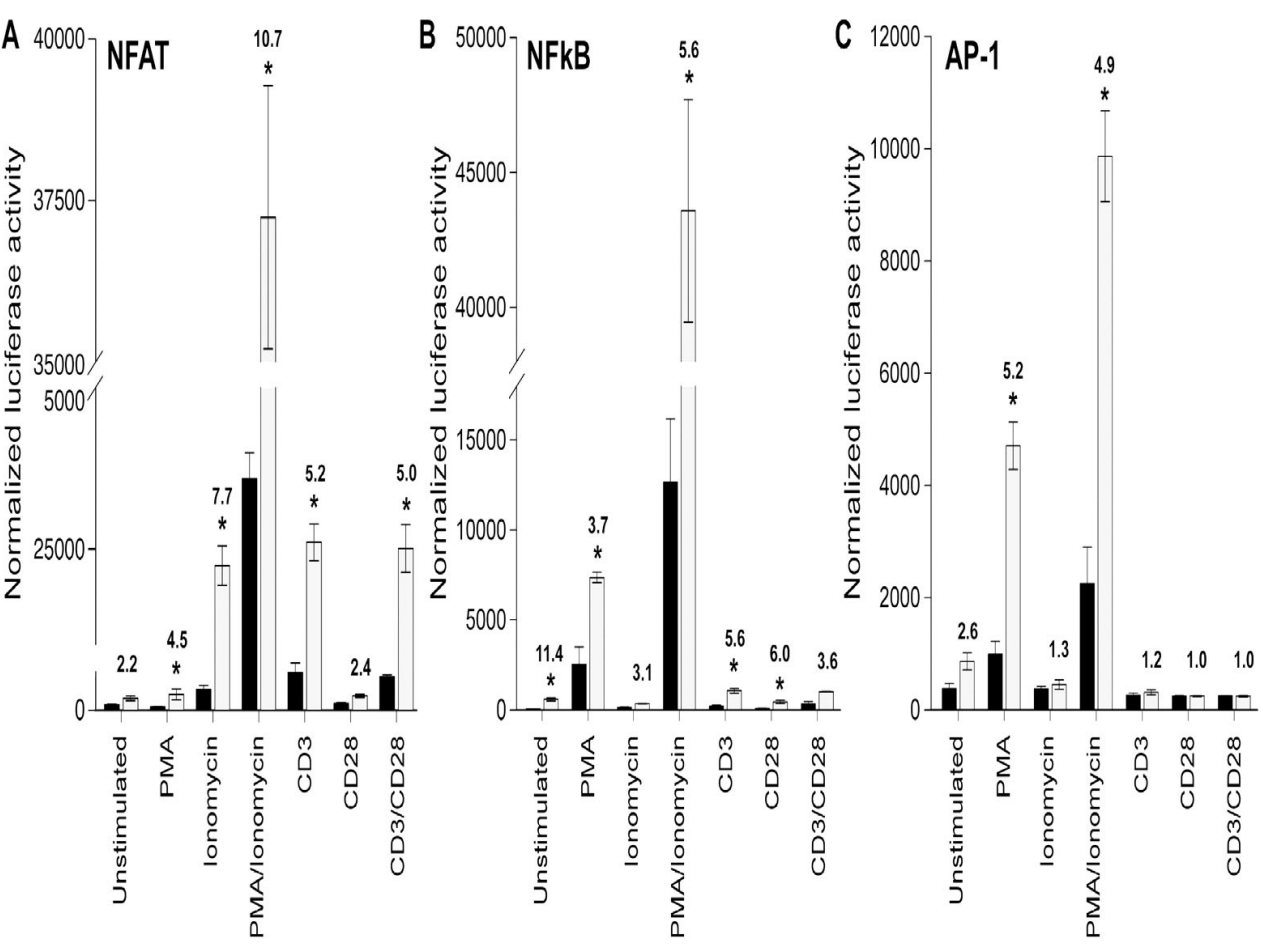
**p30^II ^activates NFAT, AP-1 and NF-κB transcriptional activity in Jurkat T lymphocytes. **Black bars indicate control and grey bars indicate p30^II^. Data points are mean of triplicate experiments. Fold increase in activity in the presence of p30^II ^is indicated above each bar. p30^II ^increased the NFAT-luc activity from 2.2 to 10.7 fold depending on co-stimulatory treatment e.g., PMA, ionomycin, CD3, CD28 etc. (A), p30^II ^increased NF-κB-luc activity from 3.1 to 11.4 fold (B) and modestly increased the AP-1 driven luciferase reporter gene activity from 1 to 5 fold in the presence of co-stimulator treatments (C).

Using luciferase reporter assays, we directly tested the ability of p30^II ^to influence NFAT, NF-κB and AP-1 driven transcription, all key transcription factors in T cell activation. Although p30^II ^expression overall resulted in a repressive pattern of gene expression, our data indicated that the viral protein selectively alters the cellular environment to promote NFAT, NF-kB and AP-1 mediated transcription in Jurkat T cells undergoing co-stimulation. We transiently co-transfected NF-κB, AP-1, or NFAT luciferase reporter plasmids and a p30^II ^expression plasmid into Jurkat T lymphocytes, and then stimulated the cells with well established co-stimulators of T cells including PMA or ionomycin or both, anti-CD3 or anti-CD28 or both. p30^II ^increased the NFAT driven luciferase reporter gene activity from 2.2 to 10.7 fold depending on co-stimulatory treatment (Fig. 4A), indicating that p30^II ^effectively enhanced NFAT driven transcription, when stimulated with ionomycin or anti-CD3. NF-κB driven luciferase reporter gene activity was increased from 3.1 to 11.4 fold, depending on co-stimulation (Fig. 4B). However, p30^II ^only modestly increased AP-1-driven luciferase reporter gene activity from 1.2 to 5.2 fold in the presence of co-stimulator treatments (Fig. 4C). Collectively, these data indicate that p30^II ^selectively promotes NFAT, NF-kB and AP-1 mediated transcription in Jurkat T lymphocytes undergoing co-stimulation and thus would be predicted to favor cell survival or influence cell activation.

## Discussion

Our study represents a comprehensive analysis of gene expression patterns influenced by a retrovirus accessory protein in T lymphocytes. Overall, this study confirmed that p30^II ^is a regulator of cellular genes, either directly or indirectly, and also identified several potential new functional roles for p30^II^. Our approach included methods to strengthen the reliability of our data by (a) use of triplicate samples and appropriate controls (b) use of multiple software for data analysis (c) minimization of nonspecific hybridization and background signals by using Affymetrix chip [44] (d) use of a well-characterized T lymphocyte system (Jurkat) and (e) verification of microarray data by semiquantitative RT-PCR in Jurkat T lymphocytes and primary CD4+ T lymphocytes (f) validation of microarray data by reporter assays, all of which were consistent with our micro array findings. Some of these findings are consistent with previous studies using gene arrays to test HTLV-1-transformed cell lines. For example, HTLV-1 infected cell lines contain low levels of caspase-4 and high levels of JUN [3] and cyclin B1 levels are low in HTLV-1 leukemic T cells [45]. Our study represents a comprehensive analysis of gene expression patterns influenced by a retrovirus accessory protein in T lymphocytes. An important caveat our approach of using gene arrays is that this method, while useful to indicate if an individual gene is increased or decreased in expression and therefore predicted to influence a cell signaling pathway, does not reveal the composite of transcription regulation in vivo. This may explain, in part, why our reporter gene data, which is more dependent upon the availability of transcription factors in total, may not directly, correlate to an individual gene expression result.

Others have used gene array approaches to study HTLV-1-related changes in gene expression. Harhaj *et al *[24] studied the gene expression in HTLV-1 mediated oncogenesis using human cDNA array analysis of normal and HTLV-1 immortalized T cells and found that the expression of a large number of genes involved in apoptosis were deregulated in HTLV-1 immortalized T cells. Subsequently, the same type of cDNA arrays were employed by De La Fuente *et al *[25] to study upregulation of a number of transcription factors in HTLV-1-infected cells, including zinc fingers, paired domains, and basic helix-loop-helix (bHLH) proteins. Gene expression profiles of fresh peripheral blood mononuclear cells (PBMC) from acute and chronic ATL patients were used to identify the genes associated with progression of ATL including a T cell differentiated antigen (MAL), a lymphoid specific member of the G-protein-coupled receptor family (EBI-1/CCR7) and a novel human homolog to a subunit (MNLL) of the bovine ubiquinone oxidoreductase complex [26]. Using NIH OncoChip cDNA arrays containing 2304 cancer related cDNA elements, Ng *et al*, 2001 [27] compared normal and Tax-expressing Jurkat T lymphocytes and identified Tax induced changes in gene expression, associated with apoptosis, cell cycle, DNA repair, signaling factors, immune modulators, cytokines, growth factors, and adhesion molecules. Recently, Affymetrix, GeneChip microarrays containing oligonucleotide hybridization probes representative of ~7000 genes were used to compare the expression profiles of normal activated peripheral blood lymphocytes to HTLV-I-immortalized and transformed cell lines [3]. In this study, by employing a gene chip representing ~33000 genes, we tested the role of p30^II ^on cellular gene expression profile of a larger number of genes. Gene expression data from cells in which exogenously expressed proteins, which may also be tagged for identification, may not represent what would occur during the natural infection. However, these patterns provide important clues for functional alterations which may occur during the viral protein expression. The "natural" or *in vivo *amount of expression of regulatory and accessory gene products encoded from the HTLV-1 pX gene region has not been clearly defined. Recent studies using RT-PCR analysis of cell lines suggests that pX ORF 1 and 2 mRNA is expressed at significantly lower amounts compared to tax/rex mRNA, full length genomic, or singly spliced envelope mRNA [46].

Expression from the IL-2 promoter requires binding of several transcription factors, including NFAT, AP-1 and NF-κB. NFAT is vital to proliferation of peripheral lymphocytes for HTLV-1 infection [47] while AP-1 is linked to the dysregulated phenotypes of HTLV-1 infected T cells [48] and malignant transformation [49]. Activation of AP-1 occurs through Tax-dependent and independent mechanisms in HTLV-1-infected T cells *in vitro *and in leukemia cells *in vivo *[48]. NF-κB is highly activated in many hematopoietic malignancies, HTLV-1 infected T cell lines and in primary ATL cells, even when Tax expression levels are low [49] and due to its anti-apoptotic activity, it is considered to be a key survival factor for several types of cancer. Ours is the first report demonstrating the ability of an HTLV-1 accessory protein to have broad modulating activities on the transcriptional activity of NF-κB, NFAT and AP-1. Further studies will be required to confirm the mechanisms of p30^II ^in T cell activation and to test the comparative role of p30^II ^expression in context to other regulators of transcription such as Tax.

We have previously reported that another HTLV-1 accessory protein p12^I ^stimulates NFAT mediated transcription, when stimulated with PMA, indicating that p12^I ^acts synergistically with Ras/MAPK pathway to promote NFAT activation and thus may facilitate host cell activation and establishment of persistent HTLV-1 infection [50]. Our data indicates that p30^II ^enhanced NFAT driven transcription significantly when stimulated with ionomycin or CD3, and therefore likely uses a different mechanism than p12^I^. To modulate NFAT driven transcription and subsequent T cell activation/signaling, it is possible that these two accessory proteins act synergistically. AP-1 is able to interact with transcriptional coactivator CBP/p300, as well as viral CREs and mediate HTLV-1 gene expression [48,51,52]. Intriguingly, we have previously reported that p30^II ^interacts with CBP/p300 at the KIX domain of CBP, influences CRE and TRE mediated transcription [18] and disrupts CREB-Tax-p300 complexes on TRE probes [21]. NF-κB and NFAT [53] are also known to interact with the transcriptional coactivator CBP/p300. Therefore, it is possible that p30^II ^modestly activates the transcriptional activity of NFAT, NF-κB and AP-1, at least in part, by its interaction with CBP/p300. In parallel, the HIV-1 accessory protein Vpr causes a modest increase in NF-κB, NFAT and AP-1 mediated transcription in a cell-cycle dependent fashion by causing G2 arrest [54]. Similar to HIV-1 Vpr, our gene array findings indicate that HTLV-1 p30^II ^expression was associated with decrease in cyclin B1 and WEE1 kinase levels, suggesting that p30^II ^expression likely cause G2 arrest and may thus modulate transcriptional activity of NFAT, NF-κB and AP-1, in a cell-cycle dependent manner. An important caveat of our data is the use of Jurkat T cells, which while representing human T cells, are IL-2 independent and transformed. Thus, differences in responsive genes expected from non-transformed T cells for the transcription factors screened in our study may be due to our cell line model.

HTLV-1 mediated interference with normal T-cell apoptosis is thought to be a mechanism of tumorigenicity [2], but specific mechanisms by which HTLV-1 infection or any particular HTLV-1 gene products influence on T-cell survival are not fully understood. Similar to the effect of HTLV-1 Tax on apoptosis related genes [24,27], we found that p30^II ^also deregulates multiple genes resulting in possible pro-apoptotic and anti-apoptotic effects. Since apoptosis is a well-known mechanism of cellular defense against viral infection, a possible role of p30^II ^in lymphocyte apoptosis might correlate with the requirement of p30^II ^in maintaining proviral loads *in vivo *[15]. Previous studies indicate that several members of the cell cycle machinery have altered expression in HTLV-1 infected cells [3]. Several recent studies have reviewed the aberrations in cell cycle caused by HTLV-1 Tax [6,55].

p30^II ^appears to regulate viral gene expression and modulate immune response. We have previously reported that, p30^II ^activated HTLV-1 LTR at lower concentrations and repressed at higher concentrations [18]. Interestingly, p30^II ^expression was associated with downregulation of lck (p56), which suppresses the HTLV-1 promoter [56] and upregulate HTLV enhancer factor, which is known to bind to LTR at a region involved in regulation of gene expression by the ets family of transcription factors [57]. Additionally, p30^II ^expression was associated with altered expression of cellular genes involved in immune modulation such as CD46, CD43, CD58, IFNγ and CD72.

## Conclusions

Overall, this study supports our earlier reports on the repressive role of HTLV-1 p30^II ^in gene expression [18,21,23] and sheds light on potential mechanisms by which p30^II ^functions in HTLV-1 replication or leukemogenesis. Our data confirmed that p30^II ^while a negative regulator of cellular genes, also influences T cell signaling, apoptosis and the cell cycle. Many of the effects of p30^II ^appear to overlap or counteract the influence of other HTLV-1 regulatory proteins like Tax or other accessory proteins such as p12^I^. It is possible that these proteins act coordinately or synergistically. We postulate that, by modulating the expression of various HTLV-1 proteins, the virus employs selective use of these viral proteins during different stages of the infection. However, since information on the expression profile of HTLV-1 proteins during stages of the infection is limited, additional studies are required to explore this possibility. Such future studies might provide new directions in the development of therapeutic interventions against HTLV-1 disorders, which are associated with immune-mediated mechanisms.

## Methods

### Lentiviral vectors and other plasmids

The plasmid pWPT-IRES-GFP was generated by cloning the internal ribosome entry site (IRES) sequence from pHR'CMV/Tax1/eGFP [58] (Gerald Feuer, SUNY, Syracuse) into pWPT-GFP plasmid (Didier Trono, University of Geneva). Subsequently, the plasmid pWPT-p30^II^HA-IRES-GFP was created by cloning the p30^II ^sequence from ACH [59] with the downstream influenza hemagglutinin (HA1) tag (Fig. 1). Sanger sequencing confirmed both the plasmids to have the correct sequence and were in frame. GFP and p30^II^HA expression were confirmed by fluorescence activated cell sorting (FACS) analysis (Beckman Coulter, Miami, FL) and western blot respectively. GFP expression from each of the plasmids was confirmed by flow cytometry (Beckman Coulter) and the p30^II^HA expression from pWPT-p30^II^HA-IRES-GFP plasmid was confirmed by western blot using mouse monoclonal anti-hemagglutinin antibody (1:1000) (Covance, Princeton, NJ) as described previously [18,21]. The plasmid pME-p30^II^HA was created by cloning p30^II ^sequence from ACH with HA1 tag, into pME-18S (G. Franchini, NIH). Other plasmids used include previously reported pRSV-βGal [18] and AP-1, NF-κB and NFAT-luciferase reporter plasmids [50].

### Recombinant lentivirus production and infection of Jurkat T lymphocytes and primary CD4+ T lymphocytes

Recombinant lentiviruses were produced by transfecting pHCMV-G, pCMVΔR8.2 and pWPT-p30^II^HA-IRES-GFP (sample) or pWPT-IRES-GFP (control) as described previously [60]. Briefly, 293T cells (5 × 10^6^) were seeded in a 10-cm dish and transfected the following day with 2 μg of pHCMV-G, 10 μg of pCMVΔR8.2 and 10 μg of pWPT-p30^II^HA-IRES-GFP or pWPT-IRES-GFP using the calcium phosphate method. Supernatant from 10 to 20 dishes was collected at 24, 48 and 72 h post transfection, cleared of cellular debris by centrifugation at 1000 rpm for 10 min at room temperature and then filtered through a 0.2 μm filter. The resulting supernatant was then centrifuged at 6,500 g for 16 h at 4°C. The viral pellet was suspended in cDMEM (DMEM containing 10% FBS and 10% streptomycin and penicillin) overnight at 4°C and the concentrated virus was aliquoted and stored at -80°C. To determine the virus titer, serial dilutions of the virus stock were used to spin infect 293T cells and 48 h post infection, eGFP expression and p30^II ^expression was measured by flow cytometry and RT-PCT respectively. Briefly, on the day before infection, 293T cells (1 × 10^5^) were seeded in a 6-well plate. The medium was removed the following day and the cells were then incubated with the diluted virus containing 8 μg/ml polybrene (Sigma, St. Louis, MO). Cells were then spin-infected by centrifugation at 2700 rpm for 1 h at 30°C, supplied with fresh medium and cultured for 48 h. Then cells were treated with trypsin (Invitrogen, Carlsbad, CA), pelleted and resuspended in D-PBS (Invitrogen) for fluorescence activity cell sorting (FACS) analysis on an ELITE ESP flow cytometer (Beckman Coulter). One × 10^6 ^cells were used to perform western blot to detect the expression of p30^II ^HA. Jurkat T lymphocytes (clone E6.1, American Type Culture Collection) were transduced with recombinant virus at multiplicity of infection of 4 in the presence of 8 μg/ml polybrene (Sigma) and spin-infected at 2700 rpm for 1 h at 25°C. Primary CD4+ T cells were extracted using dynabead CD4 positive isolation kit (Dynal Biotech, Lake Success, NY) according to manufacturer's instructions. Primary CD4+ T cells were stimulated with Phytohemagglutinin (PHA) for 48 h, transduced with recombinant virus at multiplicity of infection of 20 in the presence of 8 μg/ml polybrene (Sigma) and spin-infected at 2700 rpm for 1 h at 25°C. At 10 days post-transduction, GFP expression of controls and samples were verified to be above 90% by FACS analysis and the presence of p30^II ^mRNA expression in samples (and absence in controls) was verified by RT-PCR (Fig. 2).

### Western Immunoblot assay

Cells were lysed in buffer containing phosphate-buffered saline, 1% Nonidet P-40, 0.5% sodium deoxycholate, and 0.1% sodium dodecyl sulfate (SDS). Cell lysates were prepared by centrifugation at 14,000 rpm (Beckman) for 20 min at 4°C. Protein concentrations were determined by BCA assay (micro-BCA Protein Assay^®^, Pierce, IL). Equal amounts of proteins were mixed with Laemmli buffer (62.5 mM Tris [pH 6.8], 2% SDS, 10% glycerol, 0.2% bromophenol blue, 100 mM dithiothreitol). After boiling for 5 min, samples were electrophoresed through 12% polyacrylamide gels. The fractionated proteins were transferred to nitrocellulose membranes (Amersham Pharmacia Biotechnology) at 100 V for 1 h at 4°C. Membranes were blocked with 5% non-fat dry milk in PBS with 0.1% Tween for 16 hours, then incubated with mouse anti-HA monoclonal Ab (1:1,000) (clone 16B-12) (Covance Research Products, Princeton, NJ), for overnight at 4°C, and developed by using horseradish peroxidase-labeled secondary Ab (1:1,000) and enhanced chemiluminescence reagent (Cell Signaling Technology, Beverly, MA).

### Probe preparation and microarray analysis

According to the instructions of manufacturers, total cellular RNA was isolated from transduced Jurkat T lymphocytes using RNAqueous (Ambion, Austin, TX). To test the concentration and purity of the RNA samples, absorbance at 260 nm and 280 nm were measured and the 260/280 ratio was calculated using a spectrophotometer (Genequant, Amersham Pharmacia, Piscataway, NJ). The 260/280 ratio of all the RNA samples were between the range of 1.9–2.1. The probe preparation for GeneChip was performed according to the Affymetrix GeneChip Expression Analysis Technical Manual (Affymetrix, Santa Clara, CA). Briefly, cDNA was synthesized using genechip T7-Oligo (dT) promoter primer kit (Affymetrix) and superscript double stranded cDNA synthesis kit (Invitrogen), according to the manufacturers instructions. cDNA cleanup was done using Genechip Sample Cleanup module (Affymetrix). *In vitro *transcription was performed on the cDNA to produce biotin-labeled cRNA with ENZO RNA Transcript labeling kit (Affymetrix), according to the manufacturer's instructions. Complimentary RNA (cRNA) cleanup was performed using Genechip Sample Cleanup module (Affymetrix). The quality of total RNA and biotin-labeled cRNA of all the samples and controls were checked by calculating the ratio of absorbance at 260 nm and 280 nm (between 1.9 to 2.1) using a spectrophotometer (Genequant) and agarose gel electrophoresis. The labeled cRNA was fragmented to 50–200 nucleotides, and hybridized to U133A arrays (Affymetrix) using GeneChip^® ^Hybridization Oven (Affymetrix). Arrays were washed and stained using GeneChip^® ^Fluidics Station 400 (Affymetrix) and scanned by GeneArray Scanner (Affymetrix).

Quality control criteria evaluations done as part of the basic analysis include (1) The ratios of 3' signal to 5' signal of two housekeeping genes, beta-actin and GAPDH were between 0 and 3. (2) The hybridization controls BioB, BioC, BioD, and Cre were all present and in a linear relationship of intensity. (3) The scale factors between arrays did not vary by 3 fold. (4) The background intensity was not significantly higher than expected. (5) The percent of gene present was monitored and found to be not less than the standard 30%. To determine the quantitative RNA level, the average differences representing the perfectly matched minus the mismatched for each gene-specific probe set was calculated. Differential gene expression and comparative analysis was done using Data Mining Tool^® ^(Microarray suite 5) to identify probes with at least 1.5 fold difference in expression between control and p30^II ^and verified for cluster formation by dCHIP software [29]. The biological and molecular functional grouping of these probes was done using Gene Ontology Mining Tool (Affymetrix) [30].

### RT-PCR

One μg of RNA was converted to cDNA (Reverse Transcription system, Promega, Madison, WI) as described by the manufacturer. cDNA from 100 ng of total RNA was amplified with AmpliTaq DNA polymerase (Perkin Elmer, Boston, MA), PCR products were separated by agarose gel electrophoresis, normalized to GAPDH and quantified using alpha imager spot densitometry (Alpha Innotech, San Leandro, CA). DNA contamination was tested by performing a control with no reverse transcriptase. The PCR primers for p30^II ^were as follows: TAG CAA ACC GTC AAG CAC AG (forward) and CGA ACA TAG TCC CCC AGA GA (reverse). The PCR primers for CHP were as follows: CCC ACA GTC AAA TCA CTC GCC (forward) and ATG GTC CTG TCT GCG ATG CTG (reverse). The PCR primers for JUN-D were as follows: CTC TCA GTG CTT CTT ACT ATT AAG CAG (forward) and TTA TCT AGG AAT TGT CAA AGA GAA GATT (reverse). The PCR primers for NFATc were as follows: TTG GGA GAG ACA TGT CCC AGA TT (forward) and TCA TTT CCC CAA AGC TCA AAC A (reverse). The results were expressed as a graph. Statistical analysis was performed using Student's *t *test, P < 0.05.

### Transient transfection and reporter gene assay

Analysis of AP-1, NF-κB, and NFAT transcriptional activity in pME- and pME-p30^II^-transfected Jurkat T lymphocytes was performed as described previously [50]. Briefly, transient transfection of Jurkat T lymphocytes was done by electroporating 10^7 ^cells in cRPMI (RPMI 1640 containing 10% fetal bovine serum (FBS) and 10% streptomycin and penicillin) at 350 V and 975 μF using Bio-Rad Gene Pulser II (Bio-Rad, Laboratories, Hercules, CA) with 30 μg of pME-p30 or pME empty plasmid, 10 μg of reporter plasmid (NFAT-Luc, AP-1 Luc or NF-κB Luc), and 1 μg of pRSV-Gal plasmid or 1 ug pWPT-IRES-GFP plasmid. The transfected cells were seeded in six-well plates at a density of 5 × 10^5^/ml and were either left untreated or stimulated with 20 ng/ml of phorbol myristate acetate (PMA) (Sigma) or with 2 μM ionomycin (Sigma), or both at 6 h post-transfection, followed by incubation for 18 h prior to lysis for analysis of luciferase activity. Stimulations with anti-CD3 and/or anti-CD28 antibodies (each at 3 μg/ml) (BD Pharmingen, San Diego, CA) were carried out 18 h post-transfection. Following 8 h of stimulation, to measure luciferase activity, the cells were lysed with Cell Culture Lysis Reagent (Promega), and the cell lysates were tested for luciferase activity according to the manufacturer's protocol. Transfection efficiency was normalized by staining with 5-bromo-4-chloro-3-indolyl-beta-D-galactopyranoside (X-Gal) (Sigma) and counting β-Gal expressing cells. Transfection efficiency was also normalized by counting GFP positive cells under the fluorescence microscope. Results were expressed as mean of optimized luciferase activity (luciferase activity/percentage cells stained positive for β-Gal expression) in arbitrary light units (ALU) with standard error (SE) from a minimum of triplicate experiments. Statistical analysis was performed using Student's *t *test, P < 0.05.

## List of Abbreviations

Arbitrary light units, ALU

Phorbol myristate acetate, PMA

Fetal bovine serum, FBS

Human T-lymphotropic virus type 1, HTLV-1

Adult T cell leukemia/lymphoma, ATL

## Competing Interests

The author(s) declare that they have no competing interests.

## Authors Contributions

Bindhu Michael, Amrithraj M. Nair, Hajime Hiraragi, Lei Shen, Gerold Feuer, Kathleen Boris-Lawrie and Michael D. Lairmore have all met the definition of author as outlined by the Retrovirology journal. Each has made substantive intellectual contributions to a published study. Bindhu Michael, Amrithraj Nair, Hajime Hiraragi Gerold Feuer, Kathleen Boris-Lawrie and Michael D. Lairmore have made substantial contributions to conception and design, or acquisition of data, or analysis and interpretation of data. Lei Shen performed drafting the article or revising it critically for important intellectual content in particular sections related to biostatistical analysis. Each author has given final approval of the version to be published. Each author have participated sufficiently in the work to take public responsibility for appropriate portions of the content.

## Supplementary Material

Additional File 1 Listing of genes modulated by HTLV-1 p30^II^. Click here for file
