# Supplementary material for: Human T lymphotropic virus type-1 p30II alters cellular gene expression to selectively enhance signaling pathways that activate T lymphocytes
Source: Retrovirology. 2004 Nov 23;1:39. doi: 10.1186/1742-4690-1-39 (PMC538277; doi:10.1186/1742-4690-1-39)
Supplement: Additional File 1 — Listing of genes modulated by HTLV-1 p30II. [file 1742-4690-1-39-S1.doc]

**Table 1. Genes Modulated by HTLV-1 p30**II

| **Genbank ID** | **Description / Title** | **Gene Expression** |
| --- | --- | --- |
| **Apoptosis** | |  |
| NM021960 | myeloid cell leukemia sequence 1 | Completely turned off in all samples |
| AF017061 | cullin 5 | Completely turned off in all samples |
| NM000675 | adenosine A2a receptor | Completely turned off in all samples |
| NM004049 | BCL2-related protein A1 | Completely turned off in all samples |
| NM003806 | Harakiri | Completely turned off in 2/3 samples |
| NM001197 | BCL2/adeno E1B interacting protein 1 | Completely turned off in all samples |
| BC005034 | TSSC 3 | Completely turned off in all samples |
| AF314174 | caspase 2 | Completely turned off in all samples |
| AL050391 | caspase 4 | Completely turned off in all samples |
| Y09321 | TAF4b RNA polymerase II | Completely turned off in 2/3 samples |
| AK001291 | NCK-associated protein 1 | Completely turned off in 2/3 samples |
| NM003790 | TNF receptor superfamily, member 25 | Completely turned off in all samples |
| U70056 | seven in absentia homolog 1(Drosophila) | Completely turned off in 2/3 samples |
| AF222341 | CD 28 antigen | 1.8 fold downregulation |
| U07236 | lymphocyte specific PTK (lck) | 1.6 fold downregulation |
| AJ276888 | Mdm2 | Completely turned off in all samples |
| ^*^NM001197 | BCL2-interacting killer, BIK | Completely turned on in 2/3 samples |
| ^*^NM014430 | cell death-inducing DFFA-like effector b | Completely turned on in 2/3 samples |
| ^*^NM022094 | cell death activator CIDE-3 | Completely turned on in all samples |
| **Cell cycle** | |  |
| AL545982 | chaperonin containing TCP1, subunit 2 β | 1.7 fold downregulation |
| AA860806 | checkpoint suppressor 1 | Completely turned off in 2/3 samples |
| NM003672 | CDC14 cell division cycle 14 homolog A | Completely turned off in 2/3 samples |
| AF001362 | Janus kinase 2 | Completely turned off in all samples |
| NM003644 | growth arrest-specific 7 | Completely turned off in 2/3 samples |
| AI652662 | cytosolic BCAT-1 | Completely turned off in all samples |
| AK022408 | rab6 GTPase activating protein | Completely turned off in 2/3 samples |
| AJ277546 | WEE1 homolog | Completely turned off in all samples |
| BC005872 | histone deacetylase 6 | Completely turned off in all samples |
| AL518328 | basic leucine zipper & W2 domains 1 | 1.5 fold downregulation |
| BE407516 | cyclin B1 | 1.5 fold downregulation |
| ^*^AI347136 | telomeric repeat binding factor 1 | 1.5 fold upregulation |
| ^*^[AW341501](https://www.affymetrix.com/Portal?anlys=true&srsquery=wgetz?-id+PERMmichael4708871+-e+%5Bdbest-AccNumber:AW341501%5D|%5Bgenbank-AccNumber:AW341501%5D|%5Brefseq-acc:AW341501%5D) | A kinase (PRKA) anchor protein 8 | Completely turned on in 2/3 samples |
| ^*^NM004399 | DEAD/H box polypeptide11 | 1.8 fold upregulation |
| ^*^U04045 | mutS homolog 2 | Completely turned on in 2/3 samples |
| ^*^BG491844 | JUN | Completely turned on in all samples |
| **Transcription** | |  |
| NM000281 | TCF1 (PCBD, PCD, DCOH) | Completely turned off in all samples |
| NM003666 | basic leucine zipper nuclear factor 1 | Completely turned off in 2/3 samples |
| NM005693 | nuclear receptor subfamily1-H, member 3 | Completely turned off in all samples |
| NM012429 | SEC14-like 2 (S. cerevisiae) | Completely turned off in all samples |
| NM006186 | nuclear receptor subfamily4-A member 2 | Completely turned off in all samples |
| NM000965 | retinoic acid receptor, beta | Completely turned off in 2/3 samples |
| NM002518 | neuronal PAS domain protein 2 | Completely turned off in 2/3 samples |
| NM004527 | mesenchyme homeo box 1 | Completely turned off in all samples |
| NM014724 | zinc finger protein 305 | Completely turned off in 2/3 samples |
| NM003070 | SMARCA2 | Completely turned off in 2/3 samples |
| NM003438 | zinc finger protein 137 | Completely turned off in all samples |
| NM002167 | ID3 (HEIR-1) | Completely turned off in 2/3 samples |
| NM006079 | CITED2 (MRG1) | Completely turned off in all samples |
| M64240 | MAX protein | Completely turned off in 2/3 samples |
| U80737 | nuclear receptor coactivator 3 | Completely turned off in 2/3 samples |
| AB006572 | chromosome 19 ORF2 | Completely turned off in 2/3 samples |
| X79067 | zinc finger protein 36 L1 | Completely turned off in all samples |
| S79910 | homeo box A1 | Completely turned off in 2/3 samples |
| BC004145 | trinucleotide repeat containing 4 | Completely turned off in all samples |
| BE542323 | TONDU | Completely turned off in 2/3 samples |
| Y09321 | TAF4b RNA polymerase II | Completely turned off in 2/3 samples |
| X59740 | zinc finger protein, X-linked | Completely turned off in 2/3 samples |
| U88968 | enolase 1, (alpha) | 1.6 fold downregulation |
| NM024567 | hypothetical protein FLJ21616 | Completely turned off in 2/3 samples |
| NM013351 | T-box 21 | Completely turned off in 2/3 samples |
| AF016005 | arginine-glutamic acid dipeptide repeats | Completely turned off in 2/3 samples |
| ^*^NM003489 | nuclear receptor interacting protein 1 | Completely turned on in 2/3 samples |
| ^*^AW015313 | TBP associated factor I C | Completely turned on in 2/3 samples |
| ^*^NM005341 | GLI-Kruppel family member HKR3 | Completely turned on in 2/3 samples |
| ^*^NM004956 | ets variant gene 1 | Completely turned on in 2/3 samples |
| ^*^NM002158 | HTLV enhancer factor | Completely turned on in 2/3 samples |
| ^*^AB041834 | polyglutamine binding protein 1 | Completely turned on in 2/3 samples |
| ^*^BC000052 | peroxisome proliferative activated receptor α | Completely turned on in 2/3 samples |
| ^*^L22179 | MLLT2 (AF-4) | Completely turned on in 2/3 samples |
| ^*^AI989477 | SRY box 4 | Completely turned on in 2/3 samples |
| ^*^NM021020 | leucine zipper, putative tumor suppressor 1 | Completely turned on in 2/3 samples |
| **Translation** | |  |
| NM004564 | PET112-like (yeast) | 2 fold downregulation |
| BE138647 | translation initiation factor IF2 | Completely turned off in 2/3 samples |
| AI335509 | eukaryotic translation elongation factor 1 δ | Completely turned off in 2/3 samples |
| ^*^NM001958 | eukaryotic translation elongation factor 1 α 2 | 1.5 fold upregulation |
| **Cell Adhesion** | |  |
| BC002630 | integrin, beta 8 | Completely turned off in 2/3 samples |
| AI335208 | integrin, alpha 6 | 1.6 fold downregulation |
| NM007164 | MADCAM1 | Completely turned off in 2/3 samples |
| U02297 | selectin P ligand | Completely turned off in 2/3 samples |
| AI797281 | desmocollin 3 | Completely turned off in all samples |
| NM003622 | PPFIBP1 (liprin beta 1) | Completely turned off in all samples |
| AF244129 | CD84 (lymphocyte antigen 9) | Completely turned off in all samples |
| D28586 | CD58 antigen | Completely turned off in 2/3 samples |
| X60502 | CD43 (sialophorin) | Completely turned off in 2/3 samples |
| ^*^NM000899 | KIT ligand | Completely turned on in 2/3 samples |
| **T cell activation / signaling** | |  |
| NM002389 | CD46 | Completely turned off in 2/3 samples |
| NM001963 | epidermal growth factor | Completely turned off in all samples |
| NM005825 | RAS guanyl releasing protein 2 | Completely turned off in all samples |
| AF309082 | Protein Kinase D | 1.5 fold downregulation |
| AF074382 | IKKγ | Completely turned off in 2/3 samples |
| NM007236 | calcium binding protein P22 (CHP) | Completely turned off in all samples |
| ^*^NM003371 | vav 2 oncogene | Completely turned on in all samples |
| ^*^AF283777 | CD72 | Completely turned on in 2/3 samples |
| **Other** |  |  |
| ^*^M29383 | IFNγ | Completely turned on in 2/3 samples |

**Table footnote**: ^*^Genes up regulated by p30^II^, indicating that the signal intensity was either increased by a minimum of 1.5 fold (number = mean of fold increase on three arrays) or turned on (not expressed in controls) in at least two of the three p30^II^ expressing samples. Unmarked genes are down regulated by p30^II^, indicating that the signal intensity was either decreased by a minimum of 1.5 fold (number = mean of fold decreased on three arrays) or completely shut down in at least two of the three p30^II^ expressing samples.
